# Report of Discussions of the Mississippi Valley Dental Association, Held March 3, 1869

**Published:** 1869-08

**Authors:** 


### Proceedings of Societies.

### REPORT OF DISCUSSIONS OF THE MISSISSIPPI VALLEY DENTAL ASSOCIATION, HELD MARCH 3, 1869.

[Continued from page 293.]

Fourth Subject—Operative Dentistry.

Dr. H. A. Smith: I would suggest that large V shaped spaces that are so frequently made between the molars and bicuspids should be discarded, as they are always a source of annoyance to the patient.

Dr. McClelland: May not Dr. Smith's suggestion, if followed very closely, lead us to retain their friable edges and walls, that will be insufficient to sustain good fillings. Such thin borders and walls will invariably, sooner or later, be destroyed, and thus the object of filling be thwarted. Always make cutting enough to secure good, firm borders, and then fill and finish to them completely. Always make a very thorough examination; take sufficient time to investigate fully, and proceed under all the light attainable.

Dr. McCullum: Some time ago a case was presented to me, in which a man, by running against a bar of iron, broke off about one-third of a central incisor tooth, and split the remaining portion of the crown and root from side to side, almost its entire length. I extracted the pieces. Could anything have been done to retain this root and make it comfortable and useful?

Dr. H. A. Smith: I had a case some years ago, in which a root was split by a pivot which attached an artificial crown. I made a small gold band, and put it tightly on the end of the root, so that the two pieces were held firmly together, then replaced the crown as before, with the pivot. It stood firmly, and was valuable for several years.

Dr. SEDGWICK: Dr. McCullum did right, in my estimation, in the management of his case. There is, perhaps, less disposition now than some years ago to perform extra and difficult operations upon the teeth. Such cases are often unsatisfactory, and when so, the influence is bad. I had a case not long since in which a central incisor was broken off almost to the pulp chamber. I merely dressed it smooth and polished it, and I have no doubt it will remain so without inconvenience.

In filling proximal cavities I always make free space; and of such a form that the teeth will not come in contact again. In favorable cases I make contour fillings, but use great caution in the matter, in all cases.

Dr. A. BERRY: Deficiency in examination is a very common fault, and one from which much trouble arises. Every point in every case should pass the most searching examination.

Dr. A. M. MOORE: In filling, I use cylinders to a great extent. For forming cylinders or blocks I take, for instance, a half sheet of No. 6 foil, roll it diagonally on a No. 10 wire, then flatten the roll by passing it between the thumb and finger, then roll this strip squarely on a small broach until the cylinder is of the required size, and this will be regulated by the size of the cavity to be filled. In filling with them, the first two cylinders are placed in against the lateral walls, and then the third one fits firmly between them, and thus keying them in their position; then introduce the subsequent cylinders, observing the same principle all the way through until the cavity is full. The last gold introduced is a strip of adhesive foil. I always anneal the cylinders just before introducing them.

Dr. CUSHING: I advocate the large spaces between the teeth, and filling only flush with the lateral walls. I think this method will save the teeth more certainly than when a small space only is made, and thin walls left, and the form of the tooth restored as is too often attempted.

J. Taft: I think, as has already been expressed, that imperfect examination is one of the great faults of our profession. This examination should embrace far more than is ordinarily taken into account. All the conditions and circumstances, both those of good, and those of adverse influence should be noted. Very particular attention should be given to the conditions of the teeth, both of an original and accidental kind. The structure of the dentine, and the conformation of the teeth should receive very especial attention; all pits, fissures, and indentations should be very closely examined, also any spots that may appear in the enamel, and the proximal surfaces of all the teeth especially at or near the termination of the enamel. There is a very great variety in the minute structure of dentine. This we should carefully study.

In reference to details in operating I will refer to but one, viz: separation for filling proximal cavities. Large V shaped spaces should not, as a rule, be made between any of the teeth for filling. Separation sufficient for the operation is space enough after the filling is complete, to enable the patient to keep the parts thoroughly cleansed. Always have patients fully impressed with the importance of this matter. Examine carefully all fillings that may be in the teeth, and when defects are found they should at once be remedied; defective fillings are too often quietly passed over by many in our profession, and valuable teeth lost. In manipulating gold for filling it should not be touched with the fingers, but should be handled with pliars and napkins.

Dr. J. A. McClelland: The fees of the Dentist should be regulated by the time devoted to the patient, and hence a charge should be made for examination. Our time is often largely drawn upon for examinations and advice, and it should always be paid for.

Dr. Keely: I use the file in separating teeth just enough to secure good borders to the cavity, and for additional separation use the wedge. I exercise great care in preparing and forming the conical portion of an approximal cavity. When the cavity extends to the surface of the crown, or near it, I cut through into the cavity of decay, which gives free access into the decay; in a cavity thus formed the best facility is afforded for anchorage.

Dr. H. A. Smith: A free opening into the natural fissures, upon the crown of the tooth, in such cases, as referred to by Dr. Keely, is very important, to give strength of attachment to filling.

